# A modification to the Kuramoto model to simulate epileptic seizures as synchronization

**DOI:** 10.1007/s00285-023-01938-0

**Published:** 2023-06-17

**Authors:** José Alfredo Zavaleta-Viveros, Porfirio Toledo, Martha Lorena Avendaño-Garrido, Jesús Enrique Escalante-Martínez, María-Leonor López-Meraz, Karen Paola Ramos-Riera

**Affiliations:** 1 Facultad de Matemáticas, Universidad Veracruzana, Calle Paseo No. 112, Lote 12, Sección 2a, Villa Nueva, Nuevo Xalapa, 91097 Xalapa, Veracruz México; 2 Facultad de Ingeniería Mecánica y Eléctrica, Universidad Veracruzana, Prolongación de la Avenida Venustiano Carranza S/N. Colonia Revolución, 93390 Poza Rica, Veracruz Mexico; 3 Instituto de Investigaciones Cerebrales, Universidad Veracruzana, Dr. Luis Castelazo Ayala s/n, Industrial Ánimas, 91190 Xalapa, Veracruz México

**Keywords:** Kuramoto model, EEG signal, Synchronization of oscillators, Epileptic seizure, 34C15, 34C60, 34D06

## Abstract

The Kuramoto model was developed to describe the coupling of oscillators, motivated by the natural synchronization phenomena. We are interested in modeling an epileptic seizure considering it as the synchronization of action potentials using and modifying this model. In this article, we propose to modify this model, changing the constant coupling force for a function with logistic growth to simulate the onset and epileptic seizure level in an adult male rat caused by the administration of lithium–pilocarpine. Later, we select some frequencies and their respective amplitude values using an algorithm based on the fast Fourier transform (FFT) from an electroencephalography signal when the rat is in basal conditions. Then, we take these values as the natural frequencies of the oscillators in the modified Kuramoto model, considering every oscillator as a single neuron to simulate the emergence of an epileptic seizure numerically by increasing the synchronization value in the coupling function. Finally, using Dynamic Time Warping algorithm, we compare the simulated signal by the Kuramoto model with an FFT approximation of the epileptic seizure.

## Introduction

The Kuramoto model was proposed by Kuramoto (1984) to describe the synchronization of oscillators as a simplification of the Winfree model (Winfree 1967), taking a sinusoidal coupling. Strogatz (2000) provides an essential summary of the analysis of the model, and we can find excellent historical reviews carried out by Dörfler and Bullo (2014, 2011). The development of the Kuramoto model was motivated by natural synchronization phenomena, such as the synchronization of light pulses in fireflies or crickets chirping (Ott and Antonsen 2017), chemical oscillations (Kuramoto 1984; Acebrón et al. 2005), and the synchronization of neurons action potentials in the brain (Cumin and Unsworth 2007; Schmidt et al. 2014; Botcharova 2014), in this sense, we are interested in using it as a model for replicating epileptic seizures as synchronization of action potentials because synchronization of the action potential of neurons generates epileptic seizures (Fisher et al. 2005). Therefore, these applications to describe synchronization phenomena turn it into a practical and value model.

The International League Against Epilepsy and the International Bureau for Epilepsy have convened to define the disease as a brain disorder characterized predominantly by recurrent and unpredictable interruptions of normal brain function, denominated epileptic seizures (Fisher et al. 2005). In Jiruska et al. (2013), they establish that synchronization phenomena in epilepsy are very complex and may appear different depending on the signals measured, the spatial scale, or the considered definition of synchrony. We can find in the literature a great effort to understand and model epilepsy using mathematical tools and models, from the prediction of seizures using deep learning models to modifications of the Kuramoto model and studies on changes in the topologies of neural networks, as well as the prevention of such seizures through the study of connections and nodes or the recreation and modeling of EEG that replicate signals in *status epilepticus*. In this sense, our work seeks to follow up on these efforts to understand epilepsy (de Godoy et al. 2022; Hramov et al. 2016; Mohseni et al. 2017; Nguyen et al. 2020). We will use the Kuramoto model too, but we go to modify it and try to replicate an epileptic seizure as a synchronization phenomenon. The Kuramoto model is an approachable scenario that models the synchronized interaction of coupled phase oscillators where the coupling constant determines the level of synchronization. This coupling value is crucial for the timing; in this work, we propose changing that constant for a coupling function considering studies on the assimilation of lithium and pilocarpine used to provoke an epileptic seizure (Imran et al. 2015; Fan et al. 2020). In this way, our model tries to reflect the laboratory experience when inducing an epileptic seizure generated by the lithium–pilocarpine method. This approach is exciting since reviewed Kuramoto model literature usually considers coupling force as a constant value. On the other hand, in the reviewed texts that consider epileptic seizures and the Kuramoto model, we did not find evidence that they consider modeling the development of epileptic seizures considering an agent that promotes it (Lin and Lin 2009; Schmidt et al. 2014; Botcharova 2014). Furthermore, some texts consider variation in coupling force but model the connections between nodes and the weight of connection (Cumin and Unsworth 2007; Hramov et al. 2016). In this work, we consider the effect of the administration of lithium–pilocarpine as an agent to detonate an epileptic seizure and model the development of the seizure level with a function *K*(*t*).

We start with an electroencephalographic (EEG) signal from a rat that goes from basal conditions to an epileptic seizure. Our principal purpose is to simulate a signal with the modified Kuramoto model and compare it with the original EEG in a seizure state, trying to generate a seizure stage artificially. We propose considering a logistic function as a coupling force that changes over time to simulate the *status epilepticus* generated by the administration of lithium–pilocarpine (Imran et al. 2015; Fan et al. 2020). To do this, we take an EEG signal segment of 60 s (basal conditions) and, using the algorithm proposed in Zavaleta-Viveros et al. (2020), we obtain frequency values that we consider as the oscillator frequencies in the modified Kuramoto model, every oscillator represents a single neuron. Then, after a numerical experiments, we compare the simulated synchronized signal with an approximation of the EEG signal during epileptic seizure using the Dynamic Time Warping (DTW) algorithm.

## Proposed model

### Original Kuramoto model

The Kuramoto model proposed by Kuramoto (1984) is defined by

1 $$\begin{aligned} {\dot{\theta }}_i= \omega _i+\frac{K}{N}\sum _{j=1}^N \sin (\theta _j-\theta _i),\quad i = 1,\ldots ,N, \end{aligned}$$

where $\dot{\theta _i}$ is the rate of change of phase, $\omega _i$ the natural frequency and $\theta _i$ the phase of *i*th oscillator. $K>0$ represents the coupling strength, and *N* the number of oscillators. In the original model, *K* is considered constant, and *N* is not necessarily finite.

The model describes the synchronization of a population of coupled oscillators, each with its corresponding frequency taken from a unimodal distribution with all-to-all sinusoidal coupling. For our purposes, we consider this model for the finite-*N* case, as proposed in Strogatz (2000) and Aeyels and Rogge (2004), state of the art of this model can be founded in Dörfler and Bullo (2011, 2014). Next, we modify the constant coupling parameter of the model (1) by introducing a coupling function.

### Epilepsy and the lithium–pilocarpine induced *status epilepticus*

*Status epilepticus* is considered a non-self-limited type of epileptic seizure and the most extreme form of epilepsy (Trinka et al. 2012); this status can be induced in rats by lithium–pilocarpine administration (Jope et al. 1986; Morrisett et al. 1987a, b; Ormandy et al. 1991; Fujikawa et al. 1999). First, the pilocarpine promotes neural excitability by activation of M1 muscarinic receptors and increasing the release of acetylcholine (Jope et al. 1986; Hamilton et al. 1997; Hillert et al. 2014); later, it recruits the glutamatergic system responsible for *status epilepticus* maintenance through NMDA receptor activation (Jope et al. 1986; Ormandy et al. 1991; Naylor et al. 2013). Thus, pilocarpine promotes a brain-specific-temporal profile of neural activation, which progresses to *status epilepticus*. In Imran et al. (2015) and Fan et al. (2020), we can find and see how seizure severity evolution occurs over time. The seizure level increases gradually after the injection of pilocarpine; mainly, we consider the growth shown in Imran et al. (2015) and Fan et al. (2020) of the relationship between the time and seizure severity. The seizure level starts at 0 and grows rapidly, then slows down until it reaches a bound and holds its value, showing an S-shaped curve; then, we model this S-shaped behavior by taking a logistic function *K* as the coupling force in the Kuramoto model instead of a constant *K*. We emphasize that we want to model the effects caused by the injection in seizures after it is applied, which we model from *K*(*t*).

### Modification to the Kuramoto model

We modify the original Kuramoto model to replicate the seizure development. Experimentally, after pilocarpine injection, the rat begins to show behavioral changes due to the appearance of a seizure. The severity increases (seizure level grows) over time and displays a S-shaped curve (Imran et al. 2015; Fan et al. 2020). Considering this as a function, we can establish that it is increasing, positive, and bounded. Then, we model it mathematically with a logistic function, thus, considering the system defined in (1) with the parameter *K* as a function:

2 $$\begin{aligned} {\dot{\theta }}_i= & {} \omega _i+\frac{K}{N}\sum _{n=0}^N \sin (\theta _j-\theta _i),\ \ \ \ i \in \{1,\ldots ,N\},\nonumber \\ {\dot{K}}= & {} aK\left( 1-\dfrac{K}{C}\right) , \end{aligned}$$

where *a* is the growth rate, and *C* is the maximum coupling force (carrying capacity). In Fig. 6 we can see an example of the coupling logistic function; we will delve into that experiment later. Following the development of this model, we consider an analogous definition of the order parameter in Kuramoto (1984) and Strogatz (2000) for the case with *K* constant. Let us define the coupling parameter of phase coherence, denoted by $r=r(t)$, as

3 $$\begin{aligned} re^{i\psi }=\frac{1}{N}\sum _{j=1}^{N} e^{i\theta _j}, \end{aligned}$$

where $\psi$ is the oscillators’ average phase, the coupling parameter’s value should be between 0 and 1. When *r* takes a value close to 0, the system is incoherent; when *r* approaches 1, the system reaches coupling and behaves like a single oscillator, then $0<r<1$. For the finite case, we can calculate *r* as follows,

4 $$\begin{aligned} r=\dfrac{1}{N}\sqrt{\left( \sum _{i=1}^{N}\cos (\theta _i)\right) ^2+\left( \sum _{i=1}^{N}\sin (\theta _i)\right) ^2}, \quad i = 1,\ldots ,N. \end{aligned}$$

It is essential to understand that coupling force in the system will depend, in this case, entirely on the growth of *K*. There is a close relationship between the growth of the function *K* and the value of *r*. Because as the coupling force grows, the oscillator population begins more coherent, and *r* approaches the value of 1. Then when *K* increases, the system recruits more oscillators, and *r* grows. We can interpret this behavior as more neurons will start to synchronize action potentials; this recruits more neurons that begin to generate their action potential simultaneously and generate a *status epilepticus*.

In Chopra and Spong (2009), we find some results about coupling analysis and equilibrium conditions for the finite case, particularly demonstrating the existence and the expression for the critical coupling value $K_c$. It is the minimum coupling value that the model needs to achieve partial synchronization, that is to say, phase locking. This phase locking phase implies that some oscillators will be “locked” while others will remain moving in a “drifting” state (Strogatz 2000). For values under $K_c$, the model remains uncoupled. So we can know when the synchronization ensures. The expression determines this value is

5 $$\begin{aligned} K_c=\dfrac{N(\omega _{max}-\omega _{min})}{2\left( \sin (\gamma ) +(N-2)\sin \left( \frac{\gamma }{2}\right) \right) }, \end{aligned}$$

where

$$\begin{aligned} \gamma =2\arccos \left( \dfrac{-(N-2)+\sqrt{(N-2)^2+32}}{8}\right) . \end{aligned}$$

**Fig. 1:**
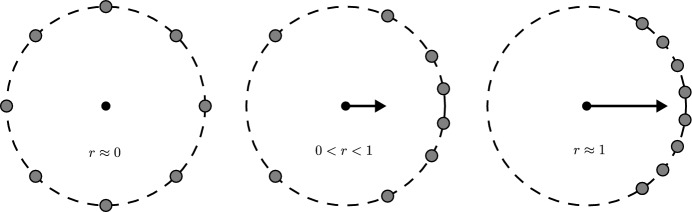
When the coupling is under $K_c$, the system remains incoherent (left arrangement) and $r\approx 0$; when the coupling overpasses $K_c$ the system begins to synchronize, a partial synchronization (middle arrangement), $0<r<1$, and when the coupling still growing up and overpasses $K_c$, the system has a locked coupling state (right arrangement) in this case $r\approx 1$

The system ensures synchronization when the coupling force reaches this partial coupling value. However, for values below $K_c$, the system does not synchronize. Then, we can interpret that an epileptic seizure may appear when the coupling force reaches that critical value. Thus, when we make the numerical analysis, we will first calculate the value of $K_c$ to have a reference value in the simulation of the epileptic seizure and ensure reaching it with coupling function *K*(*t*). As we can see in Fig. 1, the distribution of oscillators around the circle depends on the value of coupling force, as *K* growths, *r* tends to one, and the oscillators pile up in a sector of small arc length (Ha and Ryoo 2020). The bigger the coupling force is, the smaller the arc is.

We are interested in simulating qualitatively the behavior of the seizure severity occasioned by the lithium and pilocarpine. That is, our consideration over *K* wants to model the behavior of growth and the state qualitatively, not precisely a numerical analogy of the seizure stage. Then attending the considerations about $K_c$ and the model (2), we take the function *K* in (2) such that $0<K\le C$, where *C* is the maximum coupling force value that the system can reaches, in such manner $C\ge K_c$, this to achieve partial phase locking in the system, that is

6 $$\begin{aligned} \lim _{t\rightarrow \infty } K(t)= C. \end{aligned}$$

We can find exciting proposals in the literature where the coupling parameter is modified. As in Cumin and Unsworth (2007), which proposes a generalized Kuramoto system to model neural synchronization, that research proposes two modifications to the model. The first in the natural frequencies to change on time; the second in the coupling coefficient taking *K* as a function $K_{i,j}$ that depends on time “to represent time-varying coupling strengths” moreover, this function can take different values for *K* for different pairs of neurons. This purpose differentiates from ours because we model the seizure stage occasioned by the lithium–pilocarpine with a logistic function *K*(*t*); even if we consider a similar modification in the equation, the interpretation is quite different, especially conceptually and biologically. On the other hand, we take the same function for all the connections, which is different from that generalizations. Another proposal is a coupling model in Filatrella et al. (2007) and Zou and Wang (2020); they propose to incorporate a global coupling as a function in terms of r, causing the model to depend on the number of synchronized oscillators. They achieve that by taking a power-law function over the parameter *r* through an exponent $\alpha >0$; this consideration allows the study of “explosive synchronization” consisting of a sudden coupling of the oscillators, an exciting proposition but quite different to ours objectives in this work.

## The EEG signals

### Data

**Fig. 2:**
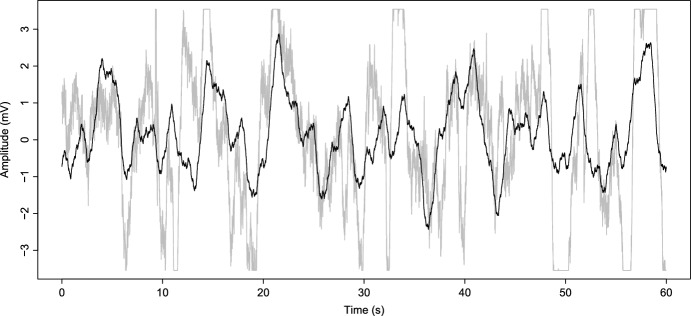
In grey the EEG signal of rat frontal cortex, basal conditions. In black the approximation obtained using the algorithm proposed in Zavaleta-Viveros et al. (2020) with fourteen components

The male Wistar rat used for this study was obtained from the facilities of the Instituto de Investigaciones Cerebrales (Universidad Veracruzana) and housed with other rats under environmental conditions of temperature and humidity (20–28$^{\circ }$C and 40–70% RH, respectively) with light–dark cycles of 12/12 h (07:00–19:00) and free access to water and food. To obtain the data, the rat was stereotaxically implanted with two nail-shaped stainless-steel electrodes on the prefrontal (PFC; $\hbox {AP} = 1.5 \,\hbox {mm}$, $\hbox {ML} = \pm \,3 \,\hbox {mm}$, relative to bregma) and parietal cortices (PC; $\hbox {AP} = -3.5 \,\hbox {mm}$, $\hbox {ML} = \pm \,3 \,\hbox {mm}$, relative to bregma) (Paxinos and Watson 2007). The surgical procedure was performed entirely in aseptic conditions and under anesthesia with isoflurane (1.5–2%, Vedco, Inc. USA). The body temperature of the rat was controlled with a temperature regulation system (FHC brand, model 41-90-D8). After the surgery, the rat was rehydrated with glucose saline (equivalent to 5% body weight, s.c.). An analgesic (Meglumine, 2.5 mg/kg s.c., for 2 days) and an antimicrobial (Enrofloxacin, 5 mg/kg s.c., for 5 days) was applied in the postoperative period. Rat underwent the experimental protocol 5 days after surgery. For seizure induction, we provided the rat with lithium chloride (3 mEq/kg i.p., Sigma) and, 24 h later, injected pilocarpine hydrochloride (30 mg/kg s.c., Sigma). A trained experimenter registered the behavioral manifestation of seizures. We digitally acquired the EEG signals using the Grass electroencephalograph Model 8-20D and ADQCH4 data acquisition software (sample frequency = 300 Hz [amplitude values acquired every 3.3 ms], sensitivity = 7 $\mu$A, band-pass filters = 1–70Hz). We continuously obtain the EEG signal in basal conditions (a baseline of 5 min before pilocarpine injection) and 1 h after pilocarpine injection in both PFC and PC. During the recording, the rat was free-moving, and we did not control their natural behavior; thus, the signal can present sudden and abrupt variations that could be due to any regular activity of the rat, for example, scratching, grooming, or sniffing. At the end of the experiment, the rat was euthanized with an overdose of sodium pentobarbital (120 mg/kg, i.p.). From this experiment we get a 2580 s long record spanning from the basal conditions evolving up to the epileptic seizure. To simulate the signal with the Kuramoto model, we consider just 1 min of signal when the rat is in basal conditions consisting of 18,000 data (60 s) and then we model a 2580 s long signal that reach synchronization. To do this, we need to obtain frequency values $\omega _i$ from the 60 s basal conditions signal using the algorithm proposed in Zavaleta-Viveros et al. (2020). This algorithm chooses frequency and amplitude from the FFT spectrum. In the example shown, they consider a $2^n$ scale: one candidate for each $n = 0,1,2,3,\ldots$ Considering the same scale with 18,000 data, we obtain a 9000 data FFT spectrum and 14 frequency values. To increase one more candidate, we need to take signals of the double longitude in every step. However, to Zavaleta-Viveros et al. (2020), the precision of approximation that we obtain in one more step has a small impact when it comes to approximating the signal; with 14 values, we have representatives of all the brain frequency bands, considering one more candidate implies a much more expensive computational process. Thus, we will select frequencies with a signal of 1-min length. We can see this signal as grey in Fig. 2.

### Obtaining frequency values for the Kuramoto model

In Zavaleta-Viveros et al. (2020), we found an algorithm capable of choosing specific frequency and amplitude values using the FFT applied to the EEG signal. Then, with these values, the algorithm constructs an approximated signal maintaining the behavior and essential information of the original one. First, we use that tool to find frequency values and use them as natural frequency values of oscillators in the Kuramoto model $(\omega _i)$. Next, we numerically solve the system of differential equations and take the amplitude value and initial phase for every frequency. Then, we get the modeled signal by doing a sum of cosine functions following the reconstruction of the FFT algorithm (Zavaleta-Viveros et al. 2020). Finally, we analyze the evolution of synchronization in this signal to give and interpret it in an epilepsy context.

We take the frontal EEG signal in basal conditions; this zone presents electrical activity during epileptic seizures generated by the effects of lithium–pilocarpine (Jope et al. 1986; Cui et al. 2020). Then we apply the algorithm developed in Zavaleta-Viveros et al. (2020) to obtain 14 values of frequency, their respective amplitude, and their initial phase. After obtaining the FFT spectrum of the signal, we have 9000 frequency and amplitude values, namely, 9000 possible frequency values for oscillators. Although, we can only consider some of them inside the Kuramoto model because we would have a model with 9000 differential equations. In this sense, the algorithm proposed in (Zavaleta-Viveros et al. 2020) is helpful because it will allow us to take up to 14 values of frequency, ensuring keep the behavior and approximation of the original signal. Furthermore, this algorithm gives us the information we need to construct the Kuramoto model: the frequency and amplitude values for reconstructing the signal. This consideration provides a considerable simplification that will allow us to perform computational experiments straightforwardly, keeping close to the behavior of the original signal. We can work with a small number of oscillators instead of handling a set of 9000 data. The chosen frequency values represent the natural frequency $\omega _i$ of an oscillator in (2) and, simultaneously, as interpretation, an oscillator represents a single neuron. We proceed to solve the system (2) numerically. Then we reconstruct the signal using the following expression (Zavaleta-Viveros et al. 2020),

7 $$\begin{aligned} {\mathcal {S}}(t) =\dfrac{A_0}{2}+ \sum _{m=1}^{n} A_m\left[ \cos \left( {\theta _m(t)}+\phi _m\right) \right] , \end{aligned}$$

where $A_m$ is the amplitude for each natural frequency $\omega _m$, $\theta _m$ the phase of oscillator, and $\phi _m$ their initial phase. In this sense, our first approach to the analysis of (2) is numerical analysis, considering the case for $N=14$ oscillators and taking the logistic function as *K*(*t*).

## Numerical simulations

In this section, we develop two numerical simulations, one for the Kuramoto model with *K* constant (1) and the other for the model proposed in the Eq. (2) to observe differences between them. For both simulations, we consider $N=14$ and the values of $\omega _i$ extracted from the 60-s EEG signal in basal conditions using the algorithm proposed in Zavaleta-Viveros et al. (2020), which are shown in Table 1.

**Table 1:** Values of the natural frequencies, amplitude values, and initial phase obtained from EEG given by the selection algorithm in Zavaleta-Viveros et al. (2020)

Oscilator	Amplitude (mV)	Frequency (Hz)	Initial phase	Brainwave type
$\omega _1$	0.1301329	$0.01{\bar{6}}$	$-\,33.85678^{\circ }$	Infra-low delta
$\omega _2$	0.04297128	$0.0{\bar{3}}$	$-\,128.76518^{\circ }$	Infra-low delta
$\omega _3$	0.326918	0.05	$-\,12.38239^{\circ }$	Infra-low delta
$\omega _4$	0.6956973	$0.11{\bar{6}}$	$119.58692^{\circ }$	Infra-low delta
$\omega _5$	0.8955179	$0.1{\bar{6}}$	$130.46331^{\circ }$	Infra-low delta
$\omega _6$	0.6738796	0.3	$-\,142.66096^{\circ }$	Infra-low delta
$\omega _7$	0.4312608	$0.5{\bar{6}}$	$-\,40.54303^{\circ }$	Delta
$\omega _8$	0.1357735	$1.1{\bar{3}}$	$-\,140.43473^{\circ }$	Delta
$\omega _9$	0.05983521	$2.{\bar{3}}$	$-\,164.73776^{\circ }$	Delta
$\omega _{10}$	0.05340884	$4.{\bar{3}}$	$-\,118.39439^{\circ }$	Theta
$\omega _{11}$	0.02188896	$8.61{\bar{6}}$	$-\,16.44028^{\circ }$	Alpha
$\omega _{12}$	0.01331891	$17.8{\bar{3}}$	$-\,120.91131^{\circ }$	Beta
$\omega _{13}$	0.009562677	$37.6{\bar{3}}$	$-\,89.89038^{\circ }$	Gamma
$\omega _{14}$	0.007953602	$119.9{\bar{3}}$	$-\,35.71958^{\circ }$	Gamma

### Original Kuramoto numerical simulation

**Fig. 3:**
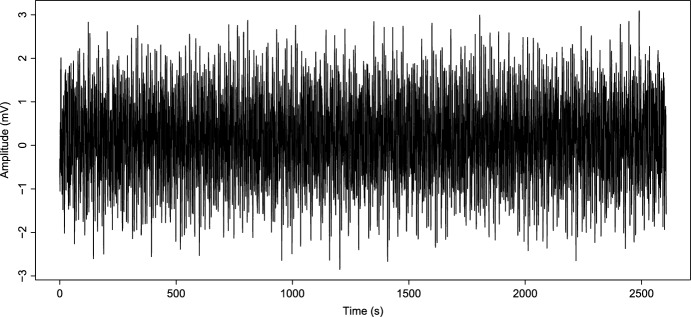
Simulated epileptic seizure as the reconstruction *S*(*t*) in (7) of the original Kuramoto model with $K=1$

**Fig. 4:**
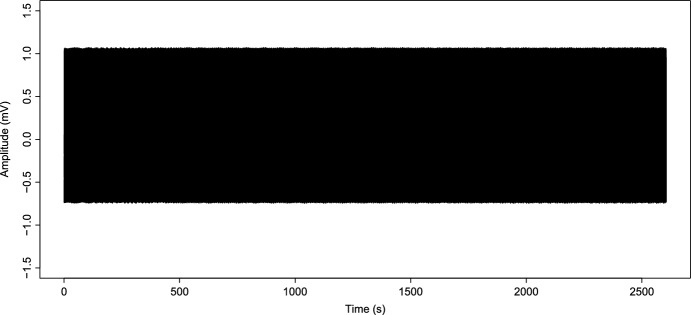
Simulated epileptic seizure as the reconstruction *S*(*t*) in (7) of the original Kuramoto model with $K=75$

We show a first simulation of the original Kuramoto model to compare with the modified Kuramoto model simulation. Assuming that $K_c=69.02359$, we simulate taking a value under and above this $K_c$, a value before coupling and a value after the system achieves phase locking. First, we take $K=1$ and simulate the signal; in Fig. 3, we can see what happens; we have a desynchronized signal that does not change his behavior through time and maintain the same behavior. In this case, we have a signal that does not present an epileptic seizure. On the other hand, in Fig. 4, we consider a value of $K=75$ above $K_c=69.02359$, as we can see, in this case, the signal shows a coupled behavior, and the signal behavior as an oscillator, as established in Strogatz (2000). This signal models the epileptic seizure.

### Modified Kuramoto numerical simulation

Next, we are interested in analyzing how the model synchronizes while *K* increases and generate a simulated signal of 2580 s. In this sense, we need to observe how the behavior of value *r* changes while *K* grows, reaches, and overpasses $K_c$. Then, we want to describe the emergence of a possible epileptic seizure by the Kuramoto model description. Finally, we will perform these numerical simulations in R software using the deSolve package and function “ode” to solve the system of ordinary differential equations and the dtw package with the dtw function to compare signals.

The frequency, amplitude, and initial phase values obtained from the EEG signal with the selection algorithm, including the Brainwave type according to Zavaleta-Viveros et al. (2020), are shown in Table 1.

Before performing the numerical analysis and establishing constant values for the function *K*(*t*), we need to calculate the value of $K_c$ for our frequency values. Then taking the $\omega _i,$ for $i=1,\ldots ,14$ given in Table 1, we get that $K_c=69.02359$, and this means that for values under 69.02359, the model can not achieve synchronization. So, we consider the logistic function *K*(*t*) from (2) with $a=0.0065$ and maximum coupling force $C=75$ to reach and exceed the partial phase locking $K_c$, thus

8 $$\begin{aligned} {\dot{K}}=0.0065K\left( 1-\dfrac{K}{75}\right) . \end{aligned}$$

**Fig. 5:**
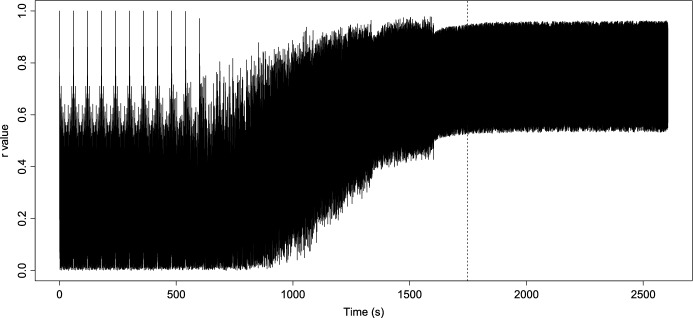
Growth of coupling parameter *r*

**Fig. 6:**
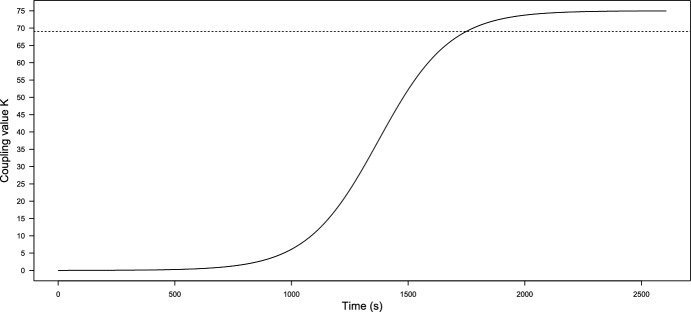
Evolution of the function *K*(*t*) over time as the logistic function (8) with $a=0.0065$ and $C=75$

**Fig. 7:**
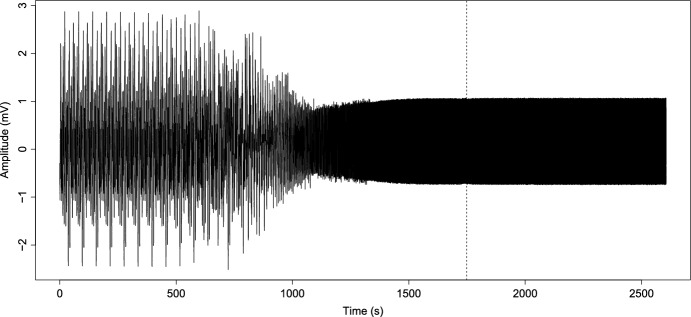
Simulated epileptic seizure as the reconstruction *S*(*t*) in (7) of the modified Kuramoto model

We can see the growth of *K*(*t*) in Fig. 6. On the other hand, taking (4), we calculate the *r*(*t*) value, and its behavior can be seen in Fig. 5. It is important to note that the function *K*(*t*) in Fig. 6 reaches the value $K_c$ around 1800 s, as well as in Fig. 5, from this value, *r* shows a regular behavior, oscillates between 0.6 and 0.9 and maintains an oscillatory behavior without variations as before reaching $K_c$. Then this implies that the model achieves phase locking and partial synchronization. On the other hand, in Fig. 7, we can see the simulated signal *S*(*t*) obtained after numerical simulation with (7). Note that around 1000 s, the signal begins to show different behavior because, at that moment, the synchronization value begins to grow (see Fig. 6). Furthermore, around 1500 s, the signal seems to become a single giant oscillator; that oscillation is the average of the phases $\psi$ (as we mentioned in (3)) and makes sense with the values observed in the graphics of *K* and *r* since, around the 1500 s is when the coupling value is reached, this suggest that, the system behaviors change, as in an epileptic seizure. Next, we compare the simulated signals with the original ones.

### Dynamic Time Warping

We consider the Dynamic Time Warping (DTW) algorithm to compare the original signal and the Kuramoto simulation. The DTW allows us to measure the similarity between two temporal sequences in time, which can vary in time and domain. This algorithm calculates an optimal match for the sequences, considering distances between all the points, creating an accumulated cost matrix, then, by a selection of the lower values, generates a path. We chose to use DTW according to study (Ding et al. 2008).

**Fig. 8:**
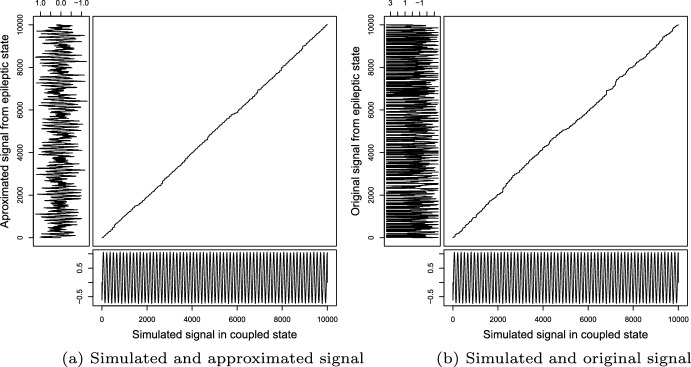
**a** In this case, we have under the horizontal the simulated signal for 14 oscillators and in vertical the reconstruction from the epileptic seizure state signal, **b** for this case, under the horizontal again we have the simulated signal for 14 oscillators and in vertical the original signal in a epileptic seizure state

We emphasize that we consider this algorithm as a qualitative measure and not as a numeric measure. Similarly, linear growth in the cumulative cost matrix path implies that the two signals are similar. Next, we will compare the simulated signals obtained under the modified Kuramoto model when synchronized with those obtained from the reconstruction with the algorithm in Zavaleta-Viveros et al. (2020) in *status epilepticus*. Figure 8b shows that comparison and path. Finally, we compare it with the original signal in *status epilepticus*. This comparison graphic of the path is in Fig. 8a.

As we can see, both path looks similar, but in the case of comparison between simulation and original signal (b), the growth has more variation. That happens because the original signal has more information and variations than the approximated signal rebuilt from 14 frequency values. Furthermore, in the case of approximation (a), the DTW path growth looks like linear growth, which implies these signals behave more similarly than the original and simulation ones.

## Discussion

We started simulating an epileptic seizure under the modified Kuramoto model taking values of frequencies from an EEG signal obtained from rats. We can observe from the DTW algorithm analysis in Sect. 4.3 that the simulated signal and the original one have similar information and behavior, in the sense of simplification of course, since we take only 14 candidates of frequency from 18,000 available in the first minute of the signal. The above represents an enormous simplification, and valuable results since the simulation behaves similarly to the real one. More precisely, we can analyze by taking reconstructions or simulations with more elements. However, increasing the number of elements (even in one) implies expensive computational costs (Zavaleta-Viveros et al. 2020). This way, 14 elements represent a good number for numerical analysis without engaging the computational cost.

**Fig. 9:**
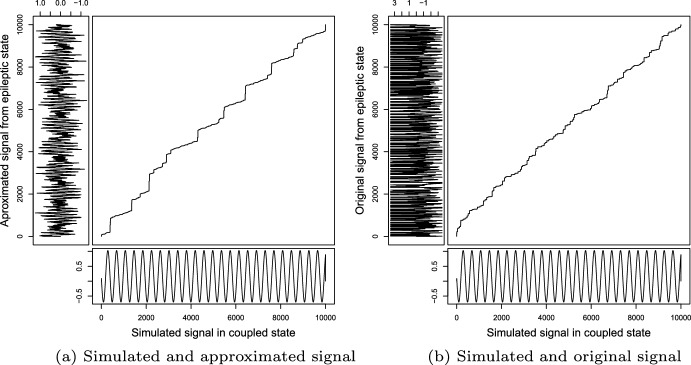
**a** In this case, we have in horizontal the simulated signal with 6 oscillators, and vertically the reconstruction from the epileptic seizure state signal, **b** for this case, in horizontal again we have the simulated signal with 6 oscillators, and vertically the original signal in a epileptic seizure state

However, to put in perspective, in Fig. 9, we show the path of the DTW for the case of 6 oscillators; we consider the first six oscillators of the Table 1 and analogously to the case of 14 oscillators, we compare the original signal in an epileptic seizure state and its approximation against the simulation when it is coupled. Note that the result of the DTW path shows a worse result when compared against the 14-oscillator path (Fig. 8). In addition, the six oscillator signal does not have all types of brainwaves and does not make biological sense (Zavaleta-Viveros et al. 2020). Additionally, note that when comparing the simulated signal of 6 oscillators against that of 14 oscillators, we can observe that the oscillation in the case of 6 oscillators is slower than that of 14. The above is due to the oscillators we have not considered having high frequencies, altering the average frequency at which the coupled signal oscillates. Thus why considering signals with all brain bands is essential.

On the other hand, it is essential to note the evolution of synchronization under the modified model in Fig. 6. We can observe that as *K* grows and reaches $K_c$ values until it reaches the maximum coupling force *C*, the value of *r* grows as well and oscillates between 0.6 and 0.9; this is due to partial coupling and the existence of “drifting” oscillators. However, it maintains a high coupling value. Likewise, before the coupling function approximates the value of $K_c$, the value of $r\approx 0$. Analogous to that behavior, the graphic of the signal Fig. 7 is irregular at the beginning. However, once the coupling value increases, the synchronization behavior provokes the simulated signal of *S*(*t*) behaviors like one oscillator with average phase $\psi$. Note that, in the case of the simulations with constant *K* (Fig. 3), evolution in coupling is not seen for $K<K_c$ and in the case of $K>K_c$ (Fig. 4), the signal is coupled from the start; in this work, is crucial to observe the evolution of the epileptic seizure, since this models the effects of lithium–pilocarpine. As we established before, we consider every oscillator as a single neuron firing an action potential. Therefore, this synchronization evolution in the signal *S*(*t*) corresponds to the simulation of the synchronization of action potentials during the development of an epileptic seizure. The fact that the simulated signal behaviors are similar to the original and approximation is a notorious achievement.

## Conclusion

Our goal in this work was to simulate an epileptic seizure with the Kuramoto model, starting from rat data in basal conditions to compare it with a signal of the epileptic seizure. The results suggest that the modified Kuramoto model was a good approach to replicating and predicting the epileptic seizure.

We proposed modifying the Kuramoto model in the coupling parameter, considering a function *K* to have logistic growth based on the behavior of seizure severity observed in Imran et al. (2015) and Fan et al. (2020). This model simulates the emergence of epileptic seizures in rats caused by the lithium–pilocarpine model. We used an algorithm proposed in Zavaleta-Viveros et al. (2020) to perform a selection and manageable numerical experiments. We observed that using the model, when the coupling force is weak, the signal has the same shape as the one obtained under the reconstruction algorithm, which can also be seen in the simulation with constant *K* when it is weak. However, as the coupling grows, the system synchronizes, and the signal behaves like one oscillator with $\psi$ frequency. We interpret this behavior as artificially replicating epileptic seizures. That is a significant achievement of our investigation because we can keep doing this analysis for more complex experiments with more oscillators to ensure precision. Furthermore, we could vary the shape or concavity in the function *K* to replicate another type of growth in coupling force and, on the other hand, permit to study of the development of epileptic seizures artificially without having to be invasive with living beings. In the future we want to compare artificial epileptic signals generated with our model with those obtained under experimental conditions in a rat. In addition, an exciting project is considering connections between different cortices to study the synchronization process considering distant brain areas; all of this analysis is under the generalization of the Kuramoto model proposed in Schmidt et al. (2014). Finally, another idea is to consider controlling the growth of coupling force by proposing a modification in *K* that models the application of a drug to avoid epileptic seizures to understand the disease better.

## Data Availability

The datasets analysed during the current study and the R code are available from the corresponding author on reasonable request.
